# ONX-0914 Induces Apoptosis and Autophagy with p53 Regulation in Human Glioblastoma Cells

**DOI:** 10.3390/cancers14225712

**Published:** 2022-11-21

**Authors:** Hsin-Han Chang, Yi-Hsuan Lin, Tzu-Min Chen, Yu-Ling Tsai, Chien-Rui Lai, Wen-Chiuan Tsai, Yu-Chen Cheng, Ying Chen

**Affiliations:** 1 Department of Biology and Anatomy, National Defense Medical Center, Taipei 114201, Taiwan; 2 Department of Nursing, Ching Kuo Institute of Management and Health, Keelung 203301, Taiwan; 3 Department of Pathology, Tri-Service General Hospital, National Defense Medical Center, Taipei 114202, Taiwan

**Keywords:** glioblastoma, ONX-0914, PR957, PSMB8, apoptosis, autophagy, p53, TMZ

## Abstract

**Simple Summary:**

ONX-0914 (PR957) is a proteasome subunit beta type-8 (PSMB8)-selective inhibitor. Previous studies have shown that inhibiting PSMB8 expression in a glioblastoma reduces tumor progression and angiogenesis. This study shows that ONX-0914 inhibits glioblastoma cell proliferation and induces cell death through apoptosis and autophagy via p53. In a mouse xenograft model, TMZ-combined ONX-0914 reduced tumor progression. This is the first study to evaluate the ONX-0914 function in glioblastomas.

**Abstract:**

Glioblastoma is believed to be one of the most aggressive brain tumors in the world. ONX-0914 (PR957) is a selective inhibitor of proteasome subunit beta type-8 (PSMB8). Previous studies have shown that inhibiting PSMB8 expression in glioblastoma reduces tumor progression. Therefore, this study aimed to determine whether ONX-0914 has antitumor effects on human glioblastoma. The results indicated that ONX-0914 treatment inhibited survival in LN229, GBM8401, and U87MG glioblastoma cells. Cell cycle analysis showed that ONX-0914 treatment caused cell cycle arrest at the G1 phase and apoptosis in glioblastoma cells. The protein expression of BCL-2 was reduced and PARP was cleaved after ONX-0914 treatment. Furthermore, the levels of p53 and phosphorylated p53 were increased by ONX-0914 treatment in glioblastoma cells. ONX-0914 also induced autophagy in glioblastoma cells. Furthermore, the p53 inhibitor pifithrin attenuated apoptosis but enhanced autophagy caused by ONX-0914. In an orthotopic mouse model, TMZ plus ONX-0914 reduced tumor progression better than the control or TMZ alone. These data suggest that ONX-0914 is a novel therapeutic drug for glioblastoma.

## 1. Introduction

Glioblastoma multiforme (GBM) is an aggressive, invasive, and undifferentiated brain tumor and has been typed as Grade IV by the WHO [1]. Unlike various solid tumors, glioblastoma overruns the surrounding normal brain tissue but seldom metastasizes [2]. Although patients receive standard radiotherapy combined with temozolomide (TMZ), the overall five-year survival rate is 9.8%. Approximately 75% of high-grade glioblastoma patients die within the first year after diagnosis [3]. Therefore, the discovery of new drugs for glioblastoma therapy is a pressing matter.

ONX-0914 (PR957) is a proteasome subunit beta type-8 (PSMB8)-selective inhibitor [4,5,6]. PSMB8 is also named large multifunctional protease 7 (LMP7) and functions as a chymotrypsin-like subunit of the immunoproteasome [4,5]. ONX-0914 is 20- to 40-fold more selective for LMP7 than LMP2 in human leukemia cells [5]. Inhibiting proteasome function induces misfolded protein accumulation, autophagy, and apoptosis [7]. In glioblastoma, PSMB8 inhibition induces apoptosis and inhibits migration and invasion through PI3K/AKT cascades [4]. PSMB8 inhibition also decreases tumor angiogenesis in glioblastomas by reducing vascular endothelial cell growth factor (VEGFA) levels [8]. Proteasome inhibition leads to cell apoptosis through increased poly ADP-ribose polymerase (PARP) and caspase 3 cleavage [4,6]. Unlike other proteasome inhibitors, ONX-0914 does not induce cell death through PARP and caspase 3 in NSCLC cells [6]. However, whether ONX-0914 causes apoptosis in GBM and the underlying mechanisms remain unclear.

P53 plays a tumor suppressor role mainly by changing the expression of numerous genes associated with cell cycle arrest, apoptosis, stem cell differentiation, and autophagy [9,10]. The Cancer Genome Atlas (TCGA) GBM project report indicates that TP53 is dysregulated in over 84% of tumors and 94% of GBM cell lines [9,11]. P53 mutation has also been observed in GBM cells [12]. Recently, p53 was shown to both suppress and enhance autophagy [13]. In GBM, p53 can inhibit the expression of O(6)-methylguanine-DNA methyltransferase (MGMT) to resist TMZ treatment [11]. Thus, restoring p53 activity in GBM may be beneficial as a GBM therapy [11]. The relationship between p53 and autophagy after ONX-0914 treatment has not been explored in GBM cells. Therefore, the aim of this study was to reveal the role of ONX-0914 in glioblastoma and its effect on apoptosis and autophagy.

## 2. Materials and Methods

### 2.1. Cell Culture

The LN229 and U87MG human glioblastoma cell lines were obtained from the American Type Culture Collection (ATCC, Manassas, VA, USA). GBM8401 was provided by Dr. Dueng-Yuan Hueng from the National Defense Medical Center (Taipei, Taiwan). All glioblastoma cell lines were maintained in RPMI 1640 (Thermo Fisher Scientific, Waltham, MA, USA) with 10% fetal bovine serum (FBS), 1% sodium pyruvate, and 1% L-glutamine (Corning, Corning, NY, USA) in a humidified atmosphere of 5% CO_2_ at 37 °C.

### 2.2. Drugs

ONX-0914, pifithrin, and TMZ were purchased from MCE (MedChem Express, Monmouth Junction, NJ, USA). MTT reagent (3-(4,5-dimethyl-2-thiazolyl)-2,5-diphenyl-2H-tetrazolium bromide), acridine orange, propidium iodide (PI), pifithrin-α, Tris-base, sodium chloride, Tween 20, and DMSO were purchased from Sigma–Aldrich (Burlington, MA, USA). Neutral buffered formalin (10%) was purchased from Leica Biosystems (Wetzlar, Germany).

### 2.3. MTT Assays

Cells were seeded in 96-well culture plates at a density of 5 × 10^3^ cells/well. To analyze cell survival in response to ONX-0914 treatment, different groups were treated for 24 h. MTT reagent was added to each well and incubated for 3 h. Then, the MTT solution was removed, followed by the addition of DMSO to dissolve the insoluble formazan. The optical absorbance was measured at 570 nm using a Synergy HTX ELISA reader (BioTek Instruments, Winooski, VT, USA).

### 2.4. Clonogenic Assays

Fifty or one hundred cells/well were seeded in 6-well plates. After 10 days, the plates were fixed with 10% neutral buffered formalin on ice and stained with Coomassie Brilliant Blue G250 (Sigma) for 30 min at room temperature. The colonies were counted and photographed after being washed with PBS and air dried.

### 2.5. Western Blotting

After drug treatment, the cells were harvested with cell lysis buffer (20 mM Tris-HCl pH 7.5, 150 mM NaCl, 1 mM Na2EDTA, 1 mM EGTA, 1% Triton, 2.5 mM sodium pyrophosphate, 1 mM beta-glycerophosphate) containing an EDTA-free phosphatase and protease inhibitor cocktail (MCE) to dissociate the cells from the plate using cell scrapers. A Q700 (Sonicator Qsonica, Newtown, CT, USA) was used to disrupt the cells and obtain the protein extract by centrifugation. Protein concentrations were measured using a protein assay kit (Bio-Rad, city, Hercules, CA, USA). Then, 5× SDS loading sample buffer (30% glycerol, 10% sodium dodecyl sulfate, 5% β-mercaptoethanol, 250 mM Tris-HCl, pH 6.8, and 0.02% bromophenol blue) was added. After 80 µg of protein samples were separated by SDS–PAGE, the proteins were transferred to nitrocellulose membranes (Bio-Rad). The membranes were blocked in BlockPROTM 1 Min Protein-free Blocking Buffer (Visual protein, Taipei, Taiwan) and incubated with primary antibodies at 4 °C overnight. The membranes were washed with TBST (20 mM Tris-base, 150 mM sodium chloride, 0.05% Tween-20) and incubated with HRP-conjugated secondary antibodies (BioLegend, San Diego, CA, USA) at room temperature. Chemiluminescent detection reagents (Bio-Rad) were applied to the blots, and the protein signals were detected by Xplorer (SPOT Imaging, Sterling Heights, MI, USA). All the antibodies used are listed in Table S1.

### 2.6. Flow Cytometry

Glioblastoma cells were seeded in 6-well plates at 4 × 10^5^ cells/well. The cells were harvested and washed with PBS after being treated for 24 h. Then, the cells were collected and fixed in ice-cold 70% alcohol. After fixation, RNase A (Thermo Fisher Scientific) was added to remove the RNA, and the cells were stained with PI (Sigma) for cell cycle distribution analysis. An APC Annexin V detection kit with 7-AAD (BioLegend) was used to identify apoptotic and necrotic cells according to the manufacturer’s instructions. Acridine orange was used in the autophagy assay. The cells were stained with 1 μg/mL acridine orange in PBS for 10 min in an incubator. The samples were analyzed by an Attune NxT Flow Cytometer (Thermo Fisher Scientific) and FlowJo software 10.4 (BD Biosciences, Franklin Lakes, NJ, USA).

### 2.7. Orthotopic Animal Model

All mouse experiments were approved by the Laboratory Animal Center of the National Defense Medical Center, Taiwan (IACUC No. 21-289). BALB/c AnN.Cg-Foxnlnu/CrlNarl mice (8 weeks old, female) were provided by the National Laboratory Animal Center (NLAC), NARLabs, Taipei, Taiwan. The mice were anesthetized with 12.5 mg/kg tiletamine-zolazepam (Zoletil 50, Virbac, Westlake, TX, USA) and 5 mg/kg xylazine (Rompun, Bayer, Leverkusen, Germany). Then, LN229 (2 × 10^5^) cells were implanted into the right cerebral hemisphere. Five days after implantation, the mice were assigned to four groups that received vehicle control, TMZ, ONX0914 (25 mg/kg), or ONX-0914+TMZ (*n* = 5 for each group). TMZ was administered via oral gavage at a dose of 5 mg/kg for seven days. The body weights of the mice were measured three times per week. The implanted tumor volume was monitored with a noninvasive In Vivo Imaging System (IVIS) (Perkin Elmer, Waltham, MA, USA) three times per week. After 15 days, the mice were sacrificed with anesthetization. The brain tissues were fixed in formalin, embedded in paraffin, and cut into serial sections.

### 2.8. HE and IHC Staining

The animal brain tissues were fixed in 10% formalin. Then, the brain tissues were sliced into 5-μm-thick sections. Histological evaluation was performed by HE staining. The expression levels of p62 in the brain tissues were examined by IHC staining. IHC staining was performed by a Ventana BenchMark ULTRA system (Roche, Basel, Switzerland). The primary antibodies were diluted in Antibody Dilution Buffer (Ventana). Antigen retrieval was performed according to the standard protocol provided by the manufacturer. Secondary goat anti-rabbit antibodies (Jackson ImmunoResearch Laboratories, West Grove, PA, USA) were used. Apoptosis was evaluated by TUNEL staining according to the manufacturer’s protocol (Roche).

### 2.9. Statistical Analysis

The data are representative of at least three independent experiments. Statistical analysis of the data was performed using one-way ANOVA. Differences between means were assessed using the unpaired Student’s *t* tests.

## 3. Results

### 3.1. ONX-0914 Inhibited the Viability of Glioblastoma Cells

**Figure 1:**
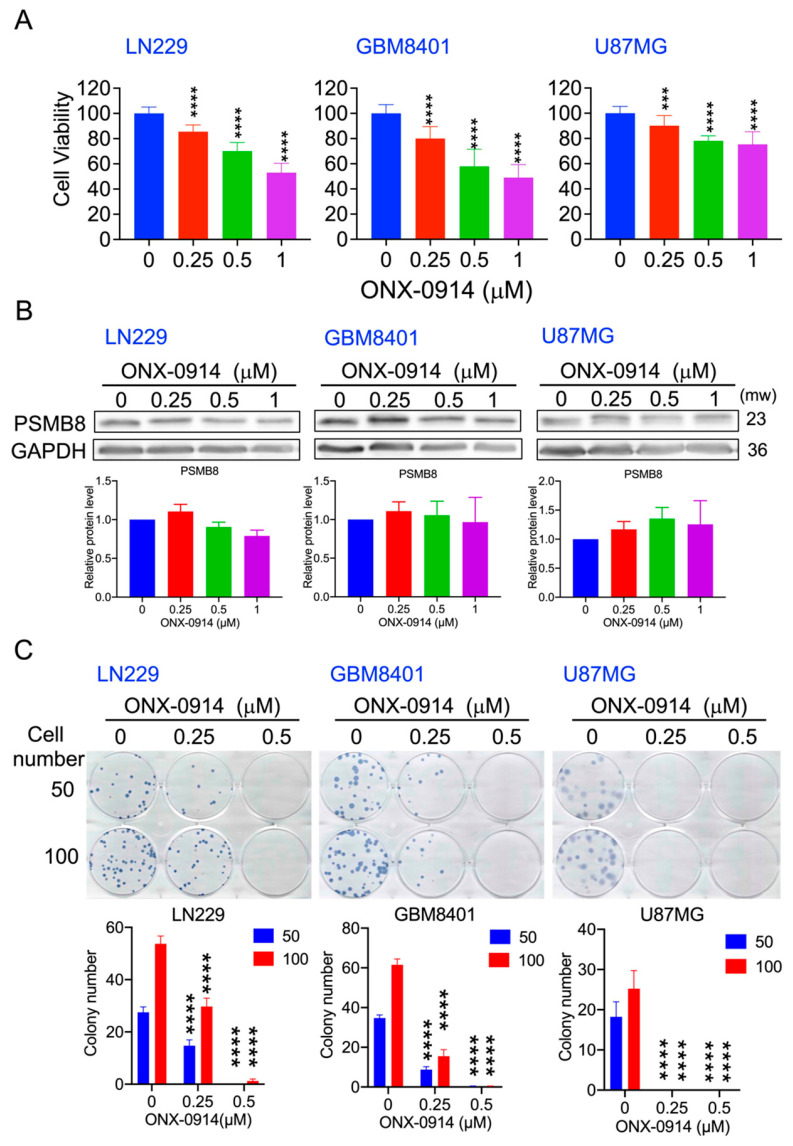
The effect of ONX-0914 on glioblastoma cell viability. (**A**) Cell viability was measured by MTT assays after glioblastoma cells were treated with ONX-0914 for 24 h. (**B**) PSMB8 protein expression was evaluated by Western blotting after ONX-0914 treatment for 24 h. The quantification is shown below. GAPDH was used as a loading control. (**C**) A total of 50 or 100 cells were seeded in 6-well dishes for the colony formation assay. ***, *p* < 0.001; ****, *p* < 0.0001 compared to the control group.

LN229, GBM 8401, and U87MG cells were treated with ONX-0914 for 24 h, and the viability of LN229, GBM8401, and U87MG cells was reduced to 53%, 75%, and 49%, respectively, after treatment with 1 mM ONX-0914 (Figure 1A). ONX-0914 did not affect the expression of PSMB8 (Figure 1B). In addition, colony formation was reduced by 0.25 and 0.5 mM ONX-0914 (Figure 1C). These results demonstrated that ONX-0914 reduced the viability of LN229, GBM 8401, and U87MG cells.

### 3.2. ONX-0914 Caused Cell Cycle Arrest in Glioblastoma Cells

**Figure 2:**
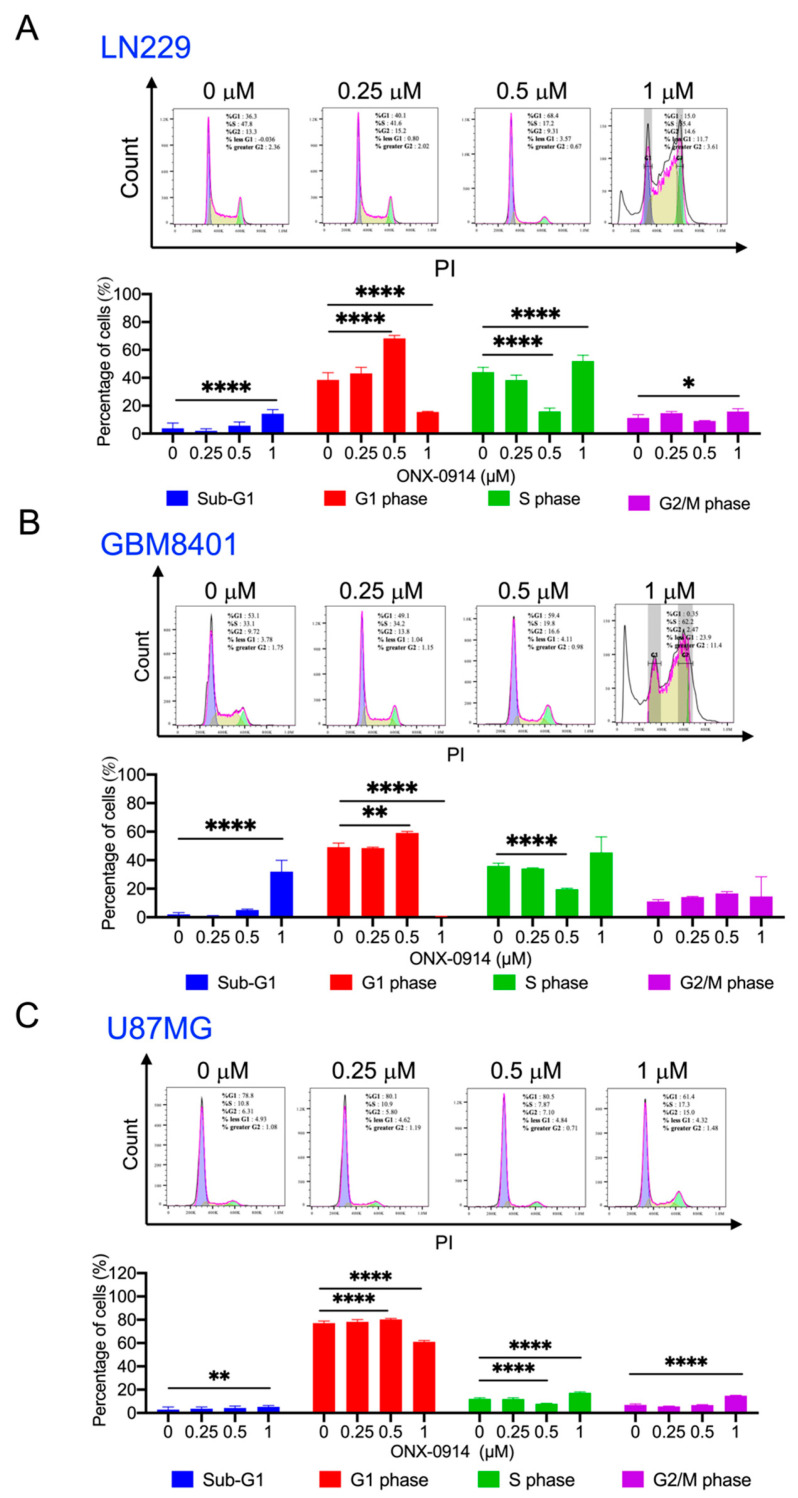
The effect of ONX-0914 on the glioblastoma cell cycle. After ONX-0914 treatment for 24 h, the cells were stained with PI. The percentage of cells in the different phases of the cell cycle is shown in the bar diagram. (**A**) LN229, (**B**) GBM 8401, and (**C**) U87MG cells. *, *p* < 0.05; **, *p* < 0.01; ****, *p* < 0.0001 compared to the control group.

LN229, GBM8401, and U87MG cells were treated with ONX-0914 for 24 h, and the cell cycle was analyzed by flow cytometry. Quantitative analysis showed that the G1 phase population of in LN229, GBM8401, and U87MG cells was increased to 68.2%, 59.5%, and 80%, respectively, after treatment with 0.5 mM ONX-0914 (Figure 2). The S phase was reduced in LN229, GBM 8401, and U87MG cells to 15.9%, 19.7%, and 7.9%, respectively, after treatment with 0.5 mM ONX-0914 (Figure 2). Moreover, ONX-0914 increased sub-G1 phase accumulation to 14.2%, 31.9%, and 5.2% in LN229, GBM 8401, and U87MG cells, respectively, after treatment with 1 mM ONX-0914 (Figure 2). These results suggested that ONX-0914 treatment arrested the cell cycle in glioblastoma cells at the G1 phase and induced apoptosis.

### 3.3. ONX-0914 Reduced BCL2 and Cleaved PARP in Glioblastoma Cells

**Figure 3:**
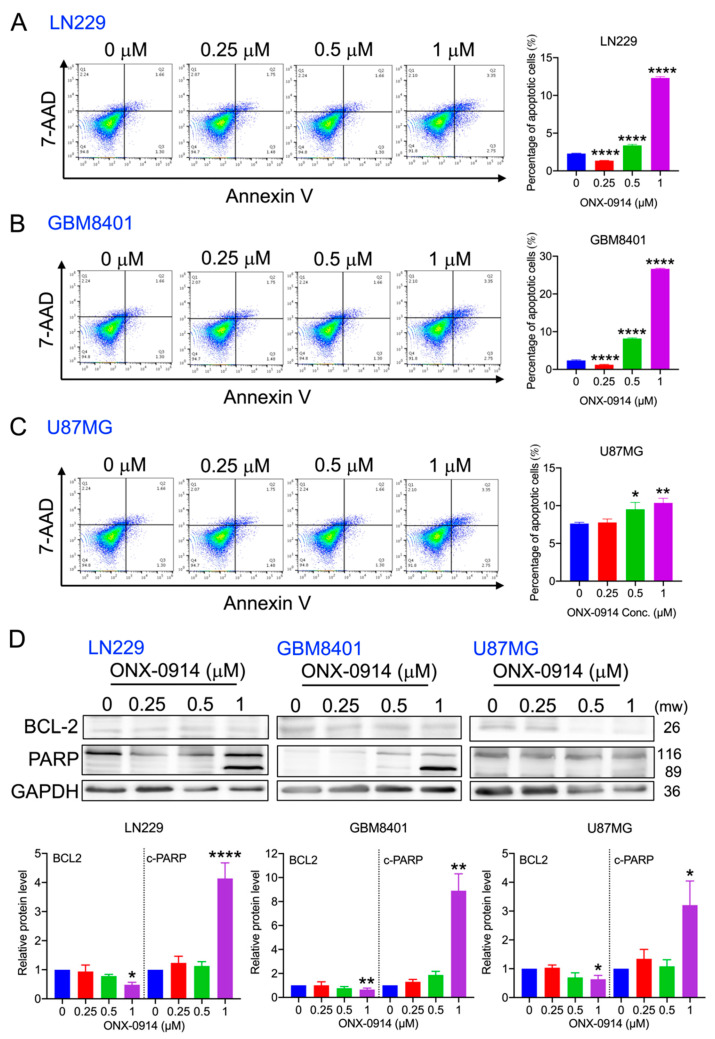
The effect of ONX-0914 on apoptosis in human glioblastoma cells. The percentage of apoptotic cell death was measured using Annexin V-APC/7-AAD staining. The percentage of apoptotic cells is shown in the bar diagram. (**A**) LN229, (**B**) GBM 8401, and (**C**) U87MG cells. (**D**) BCL-2 and cleaved PARP (c-PARP) protein expression was analyzed after ONX-0914 treatment for 24 h. GAPDH was used as a loading control. The lower panels show the quantitative results. *, *p* < 0.05; **, *p* < 0.01; ****, *p* < 0.0001 compared to the control group.

Due to the reduction in survival and sub-G1 phase accumulation in glioblastoma cells after ONX-0914 treatment, apoptosis was analyzed by Annexin V and 7-AAD staining. The results showed that ONX-0914 (1 mM) induced apoptotic cell death at rates of 12.2%, 26.6%, and 10.3% in LN229, GBM 8401, and U87MG cells, respectively (Figure 3A–C).

Apoptotic-related proteins were analyzed by Western blotting. The results showed that BCL-2 was reduced after 1 mM ONX-0914 treatment, whereas cleaved PARP was increased significantly in the three cell lines (Figure 3D). Based on these results, ONX-0914 treatment resulted in cell apoptosis through BCL-2 downregulation and PARP cleavage in glioblastoma cells.

### 3.4. ONX-0914 Induced p53 Phosphorylation and Expression in Glioblastoma Cells

**Figure 4:**
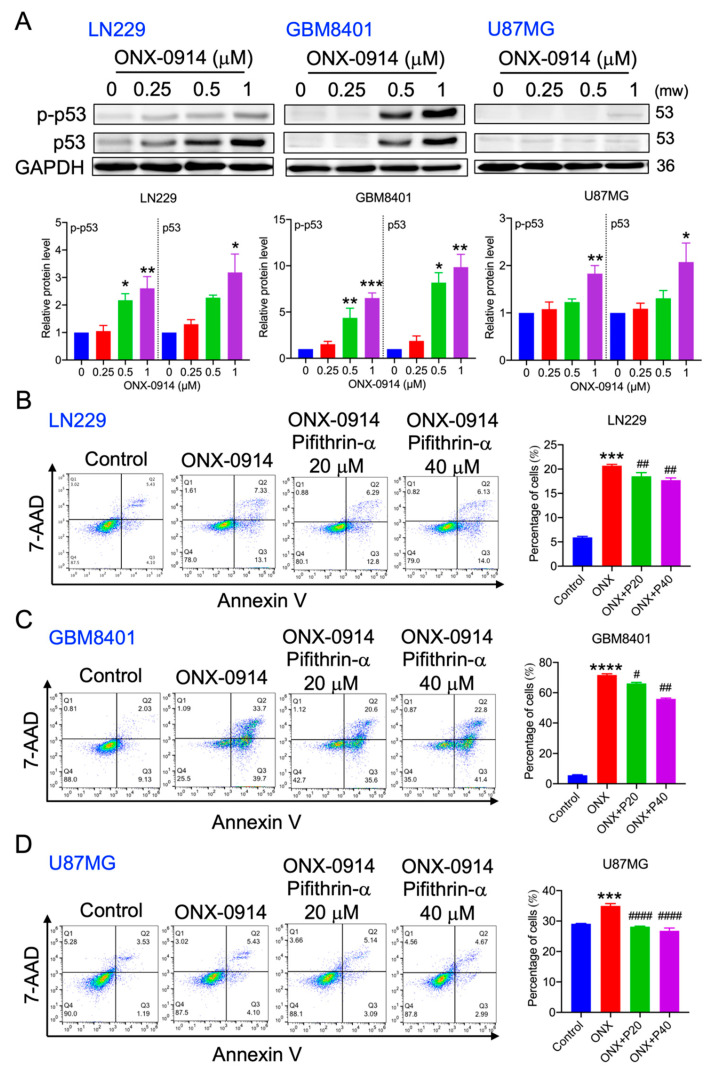
The effect of ONX-0914 treatment on p-p53, p53, and apoptosis. (**A**) Phosphorylated p53 and total protein levels were analyzed by Western blotting. GAPDH was used as a loading control. The lower panels show the quantitative results. Glioblastoma cells were treated with 1 mM ONX-0914 for 24 h after being pretreated with pifithrin for 2 h. Apoptotic cell death was measured using Annexin V-APC/7-AAD staining. The percentage of apoptotic cells is shown in the bar diagram in the right panel. (**B**) LN229, (**C**) GBM8401, and (**D**) U87MG cells. *, *p* < 0.05; **, *p* < 0.01; ***, *p* < 0.001; ****, *p* < 0.0001 compared to the control group. #, *p* < 0.05; ##, *p* < 0.01; ####, *p* < 0.0001 compared to the ONX-treated group.

p53 has been reported to manage apoptosis and autophagy in cancer cells [14]. The p53 expression was upregulated in LN229 and GBM8401 cells compared to human astrocytes (Figure S1). After ONX-0914 treatment, p53 protein expression and phosphorylation were analyzed by Western blotting. As shown in Figure 4A, phosphorylated and total p53 expression was induced 2.6-, 6.6-, and 4.6-fold in LN229, GBM 8401, and U87MG cells, respectively, compared to the control group. Moreover, total p53 expression was induced 3.18-, 9.8-, and 2.1-fold in LN229, GBM8401, and U87MG cells, respectively, compared to the control group (Figure 4A). In addition, pretreatment with the p53 inhibitor pifithrin-α significantly reduced ONX-0914-induced apoptosis to 17.0%, 54.7%, and 25.3% in LN229, GBM8401, and U87MG cells, respectively, compared to that in the ONX-0914 groups (18.9%, 70.5%, and 33.6%, respectively) (Figure 4B–D). Accordingly, p53 activation contributed to apoptosis induction during ONX-0914 treatment.

### 3.5. ONX-0914 Induced Autophagy in Glioblastoma Cells

**Figure 5:**
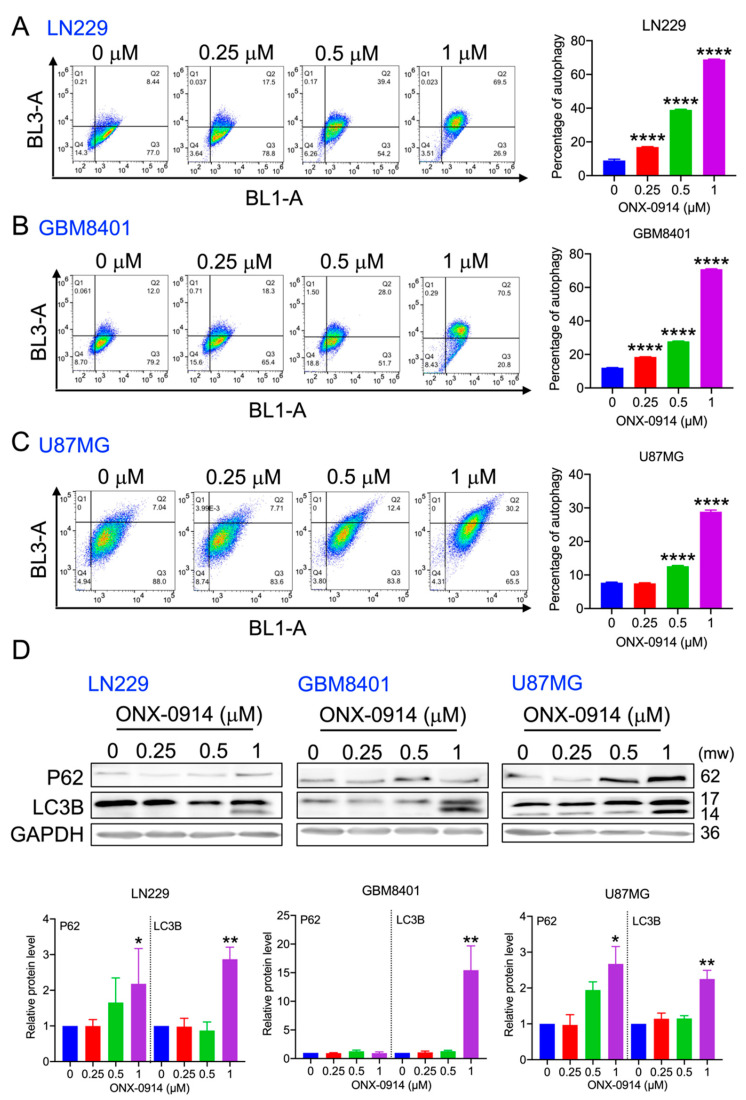
The effect of ONX-0914 on autophagy. The cells were stained with acridine orange for 10 min in the incubator. The percentage of autophagic (**A**) LN229, (**B**) GBM8401, and (**C**) U87MG cells is shown. The right panel shows the quantification. (**D**) The autophagy-related proteins P62 and LC3B were evaluated using Western blotting. GAPDH was used as a loading control. *, *p* < 0.05; **, *p* < 0.01; ****, *p* < 0.0001 compared to the control group.

Apoptosis and autophagy are crucial for maintaining homeostasis in cells [15]. LN229, GBM 8401, and U87MG cells were treated with ONX-0914 for 24 h and then stained with acridine orange. In LN229 cells, 0.25 and 0.5 mM ONX-0914 increased autophagy to 16.96% and 38.9%, respectively (Figure 5A). In GBM8401 cells, 0.25 and 0.5 mM ONX-0914 increased autophagy to 18.5% and 27.8%, respectively (Figure 5B). In U87MG cells, 0.5 mM ONX-0914 increased autophagy to 12.5% (Figure 5C). In addition, acridine orange staining was increased to 68.9, 70.8, and 28.8% in LN229, GBM 8401, and U87MG cells, respectively, after 1 mM ONX-0914 treatment (Figure 5A–C).

Moreover, autophagy-related protein expression in glioblastoma cell lines was analyzed by Western blotting. p62 was increased by 1 mM ONX-0914 in LN229 and U87MG cells compared to that in the control group (Figure 5D). In addition, LC3B-II was increased 2.8-, 15.4-, and 2.2-fold in LN229, GBM8401, and U87MG cells, respectively, compared to that in the control group (Figure 5D). These results suggested that ONX-0914 induced autophagy and LC3B-II in LN229, GBM8401, and U87MG cells.

**Figure 6:**
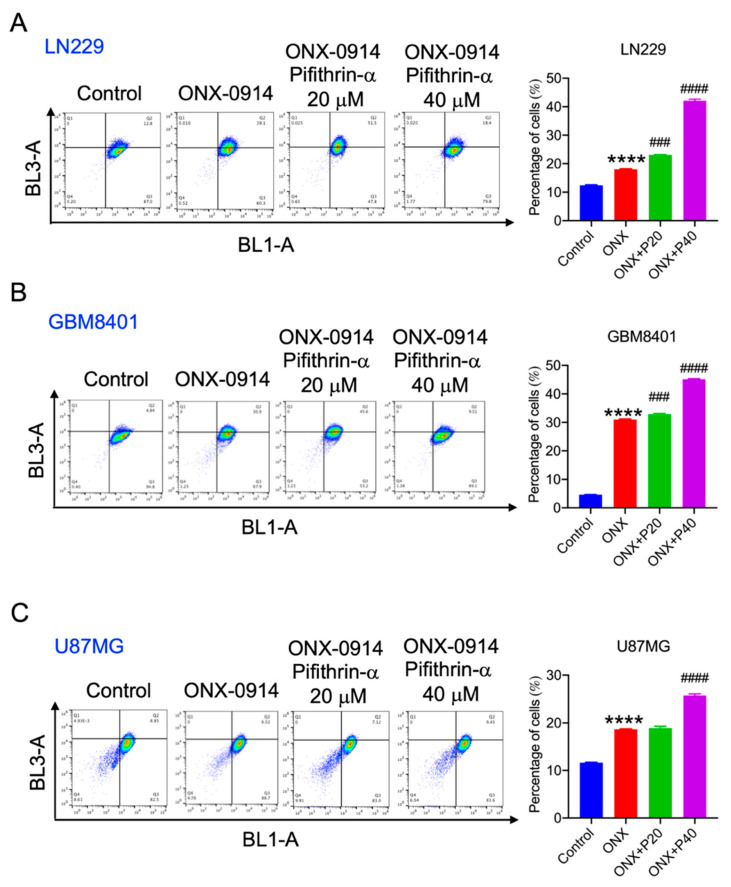
The effect of a p53 inhibitor on ONX-0914-induced autophagy in glioblastoma cells. Autophagy was detected using acridine orange staining. The flow cytometry results are shown in the dot diagram. The percentage of autophagic cells is shown in the bar diagram in the right panel; (**A**) LN229, (**B**) GBM8401, and (**C**) U87MG cells. ****, *p* < 0.0001 compared to the control group. ###, *p* < 0.001; ####, *p* < 0.0001 compared to the ONX-treated group.

Next, the relationship between p53 and autophagy was investigated. Acridine orange staining showed that pifithrin-α significantly increased autophagy in LN229, GBM8401, and U87MG cells (Figure 6).

### 3.6. TMZ Combined with ONX-0914 Inhibited Tumor Progression in Glioblastoma Cells in a Mouse Model

**Figure 7:**
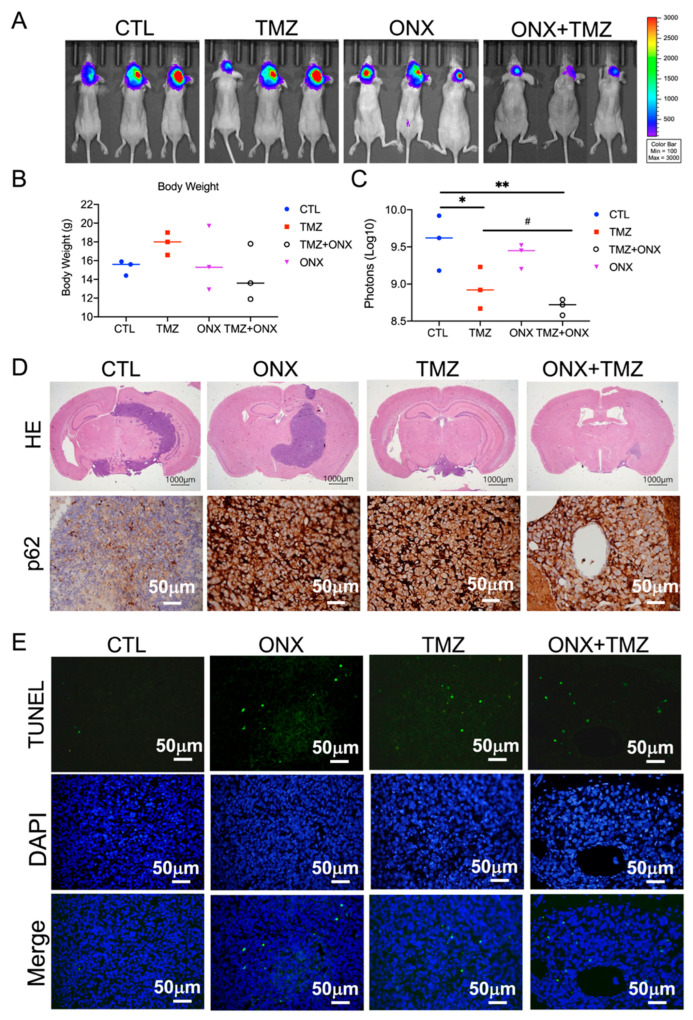
The effect of ONX-0914 on tumor progression in an orthotropic human glioblastoma xenograft model. LN229 human glioblastoma cells were implanted into nude mice. Placebo (CTL), temozolomide (TMZ), ONX-0914, and TMZ plus ONX-0914 were then administered every day for 15 days. (**A**) The in vivo bioluminescent imaging analysis of the different treatments were acquired by the In Vivo Imaging System (IVIS). (**B**) The body weight was measured and compared to the placebo group. (**C**) The luminescent intensity of photons emitted from each animal was quantified. (**D**) Representative image showing H&E and p62 staining of the xenograft orthotropic brain. (**E**) Apoptosis was measured using TUNEL and counterstaining with DAPI. *, *p* < 0.05; **, *p* < 0.01 compared to the placebo group. #, *p* < 0.05 compared to the TMZ group.

LN229-Luc2 cells were developed to monitor tumor progression in a previous study [16]. Tumor progression was measured based on the bioluminescence imaging (BLI) value. LN229-Luc2 cells were implanted into nude mice to evaluate the effect of ONX-0914 on glioblastoma (Figure 7A). After drug administration, the body weights were not significantly different in each group (Figure 7B). Treatment with TMZ combined with ONX-0914 caused significant tumor regression compared to TMZ alone (*p* = 0.0073 and *p* = 0.0383 compared to the placebo group) (Figure 7C). H&E staining showed a reduction in tumor size in the TMZ plus ONX-0914 group (Figure 7D). Moreover, p62 expression and TUNEL levels were induced by ONX-0914 (Figure 7E). These results revealed that autophagy and apoptosis may be involved in ONX-0914-mediated inhibition of tumor progression in vivo.

## 4. Discussion

Proteasomes are large multi-subunit proteolytic complexes in the ubiquitin–proteasome system (UPS) that play a crucial role in cellular protein homeostasis [17,18]. Therefore, the UPS is a prominent regulator of various cellular procedures, including but not restricted to, cell survival, cell cycle progression, gene transcription, antigen presentation, and DNA restoration [18]. Inhibiting the proteasome results in anti-proliferative and pro-apoptotic effects in glioblastoma cells [18]. However, it is unknown precisely why tumor cells are more sensitive to proteasome inhibitors than normal cells [19]. One potential reason stems from several studies that revealed that proteasomal function was further activated in tumors, which is essential for malignant tumor cells [19]. In a siRNA screening study that determined relevant genes associated with GBM patient survival, 22% (12/55) were members of the 20S and 26S proteasome subunits [19]. ONX-0914 was reported to significantly reduce the growth of acute lymphoblastic leukemia tumors in vitro and in vivo [17,20]. Proteasome inhibitors that interfere with the activity of the UPS may disrupt various cellular processes and stop proliferation or induce cell death [18]. According to our results, ONX-0914 inhibited the survival rate and colony formation of three human glioblastoma cell lines. Moreover, ONX-0914 induced apoptosis with PARP and caspase 3 cleavage in human glioblastoma cells.

The UPS and autophagy-lysosomal pathway (ALP) are two crucial protein degradation mechanisms within cells [7]. MG132 induces glioblastoma cell apoptosis through caspase 3 activation [21,22]. A previous study revealed that MG132 induced cell death and apoptosis in human SHG-44 glioma cells [23]. The autophagy inhibitor 3-methyladenine (3-MA) and MG-132 inhibits cell death, autophagy, and cell cycle arrest at G2/M in the SHG-44 cell line [23]. The inhibition of autophagy enhances apoptosis in glioma cell lines [24]. Bortezomib and TRAIL (tumor necrosis factor-related apoptosis-induced ligand) induce cell death and reduce colony formation in U87MG and T98G glioblastoma cell lines [25]. Bortezomib inhibits cell proliferation and colony formation [26]. According to the results, ONX-0914 induces autophagy and apoptosis in glioblastoma cells.

Inhibition of the proteasome may also increase the stability of tumor suppressor proteins such as p27 and p53, which reduces proliferation [18]. An earlier study revealed that the proteasome inhibitor lactacystin caused the accumulation of p53 and autophagy in primary ventral mesencephalic neurons [7]. Caspase 3/7 activity is inhibited by pifithrin-α in the D54 human glioblastoma cell line [22]. In contrast, inhibition of p53 by pifithrin-α or small interfering RNA (siRNA) attenuated autophagy induction and improved protein aggregation [7]. MG132 induces p53 expression in the D54 glioblastoma cell line [22]. MG132 and bortezomib induce PARP and caspase3 cleavage in the lung cancer cell lines H441, H460, and A549 [6]. ONX-0914 does not induce cleaved PARP and caspase 3 in the lung cancer cell lines H441, H460, and A549 [6]. In head and neck squamous cell carcinoma (HNSCCS), carfilzomib and ONX-0912 (another PSMB8 inhibitor) induce apoptosis and autophagy [27]. However, ONX-0914-induced apoptosis was inhibited by the p53 inhibitor pifithrin-α, whereas autophagy was enhanced by pifithrin-α treatment in our study. A previous study revealed that T98G with mutant p53 was the most sensitive to bortezomib, as determined by MTT assays [26]. Glioma cell lines with p53 mutation were more sensitive to TMZ than wild-type glioblastoma cell lines [11,28,29]. According to the cell proliferation results, LN229 and GBM8401 cells with mutant p53 were more sensitive to ONX-0914. Thus, we suggest that ONX-0914 causes apoptosis through p53 activation in glioblastoma cell lines.

Pifithrin-a has been reported to block the transcription activity and phosphorylated status of p53 [30,31]. However, pifithrin-a also protects both p53 wild-type and p53-deficient colon cancer cells from irradiation-induced apoptosis [31]. This p53-independent effect of pifithrin-a may be caused by the involvement of mitochondria and cyclin D1. Therefore, the inhibition of ONX-0914-induced apoptosis by pifithrin-a might display a p53-independent manner in human glioblastoma cells. Additionally, although the background of p53 is different in LN229 and U87MG cells, ONX-0914 exerted apoptosis and autophagy in human glioblastoma cells. The ONX-0914-induced apoptosis was declined and -autophagy was enhanced whereas pifithrin-a was applied. P53 has been reported to manage apoptosis and autophagy in cancer cells [14]. Moreover, cytoplasmic p53 inhibits autophagy by interacting with Beclin-1 in embryonal carcinoma cells [32]. However, tumors with variants p53 or without p53 can still inhibit autophagy [33]. Therefore, whether ONX-0914 modified apoptosis and autophagy depended on the p53 status needs further investigation.

Present GBM treatments include surgery, radiation therapy, and chemotherapy [11]. Even with these treatments, the median survival of GBM patients after diagnosis is only ~15 months [9]. Various setbacks in recent years have plagued drug development for glioblastoma [18]. Since the first proteasome inhibitor bortezomib was approved by the FDA for treating multiple myeloma and mantle cell lymphoma, the development of anticancer medicines targeting the proteasome and the components of the UPS has been the most vibrant field of study [34]. Although clinical tests reveal that proteasome inhibitors are not significantly efficient in treating gliomas, second-generation proteasome inhibitors have been produced with improved pharmacokinetic properties [19]. According to the results, TMZ combined with ONX-0914 inhibited tumor progression in a mouse xenograft model.

In this study, we clarified that the PSMB8 inhibitor, ONX-0914, arrested cell cycle, and induced apoptosis and autophagy in three human glioblastoma cells. However, there are limitations in this study. First, we did not apply ONX-0914 in patient samples, as demonstrated by Min, Lingzhao, et al., where ONX-0912 (another PSMB8 inhibitor) promotes apoptosis and autophagosome accumulation to inhibit proliferation of glioma cells [35]. Second, a mouse xenograft model with an intraperitoneal injection of ONX-0914 combined with oral chemotherapy drug TMZ was used in this study. The metabolic pathway of ONX-0914 in the brain is not explored yet. We believed this study provides a strong rationale for testing PSMB8 inhibitors in patient samples and ultimately in clinical trials in GBM.

## 5. Conclusions

In conclusion, the PSMB8 inhibitor ONX-0914 reduced glioblastoma progression by inducing cell cycle arrest, apoptosis, and autophagy.

## Data Availability

The data presented in this study are available on request from the corresponding author. Data may be available upon request to interested researchers.

## Supplementary Materials

The following supporting information can be downloaded at: https://www.mdpi.com/article/10.3390/cancers14225712/s1. Figure S1: P53 expression in human astrocytes and glioblastoma cells. Table S1: All the antibodies used are listed in it. File S1: Original blots.
