# Supplementary material for: ONX-0914 Induces Apoptosis and Autophagy with p53 Regulation in Human Glioblastoma Cells
Source: Cancers (Basel). 2022 Nov 21;14(22):5712. doi: 10.3390/cancers14225712 (PMC9688407; doi:10.3390/cancers14225712)
Supplement: Supplementary file 1 [file cancers-14-05712-s001.zip › cancers-2016896-supplementary.pdf]

Supplementary Materials: ONX-0914 induces apoptosis and autophagy via p53 induction in human glioblastoma cells

Hsin-Han Chang, Yi-Hsuan Lin, Tzu-Min Chen, Yu-Ling Tsai, Chien-Rui Lai, Wen-Chiuan Tsai, Yu-Chen Cheng and Ying Chen

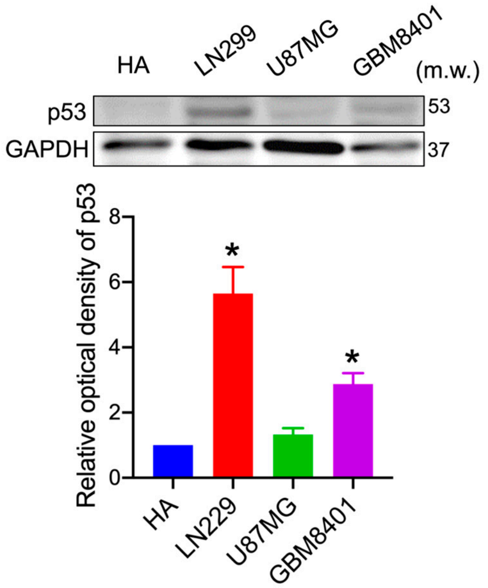

Figure S1. The p53 protein expression in human glioblastoma cells. HA: cell lysate of human astrocytes. * $p < 0.05$ compared to HA group. $n = 3$.

Table S1. The information of antibody.

Name	Species	Brand	Cat
GAPDH	Rb	CST	5174S
PSMB8	Rb	CST	13635S
BCL-2	Rb	CST	4223S
PARP	Rb	CST	9532S
p-p53	Rb	CST	9284S
p53	Rb	CST	9282S
p62	MS	Santa Cruz	SC-28359
LC3B	Rb	CST	3868S
p-AKT (T308)	Rb	CST	13038S
AKT	Rb	CST	4691S
p-mTOR	Rb	CST	5536S
mTOR	Rb	CST	2972S
