# Supplementary figures and images for: ONX-0914 Induces Apoptosis and Autophagy with p53 Regulation in Human Glioblastoma Cells
Source: Cancers (Basel). 2022 Nov 21;14(22):5712. doi: 10.3390/cancers14225712 (PMC9688407; doi:10.3390/cancers14225712)

Fig 1B

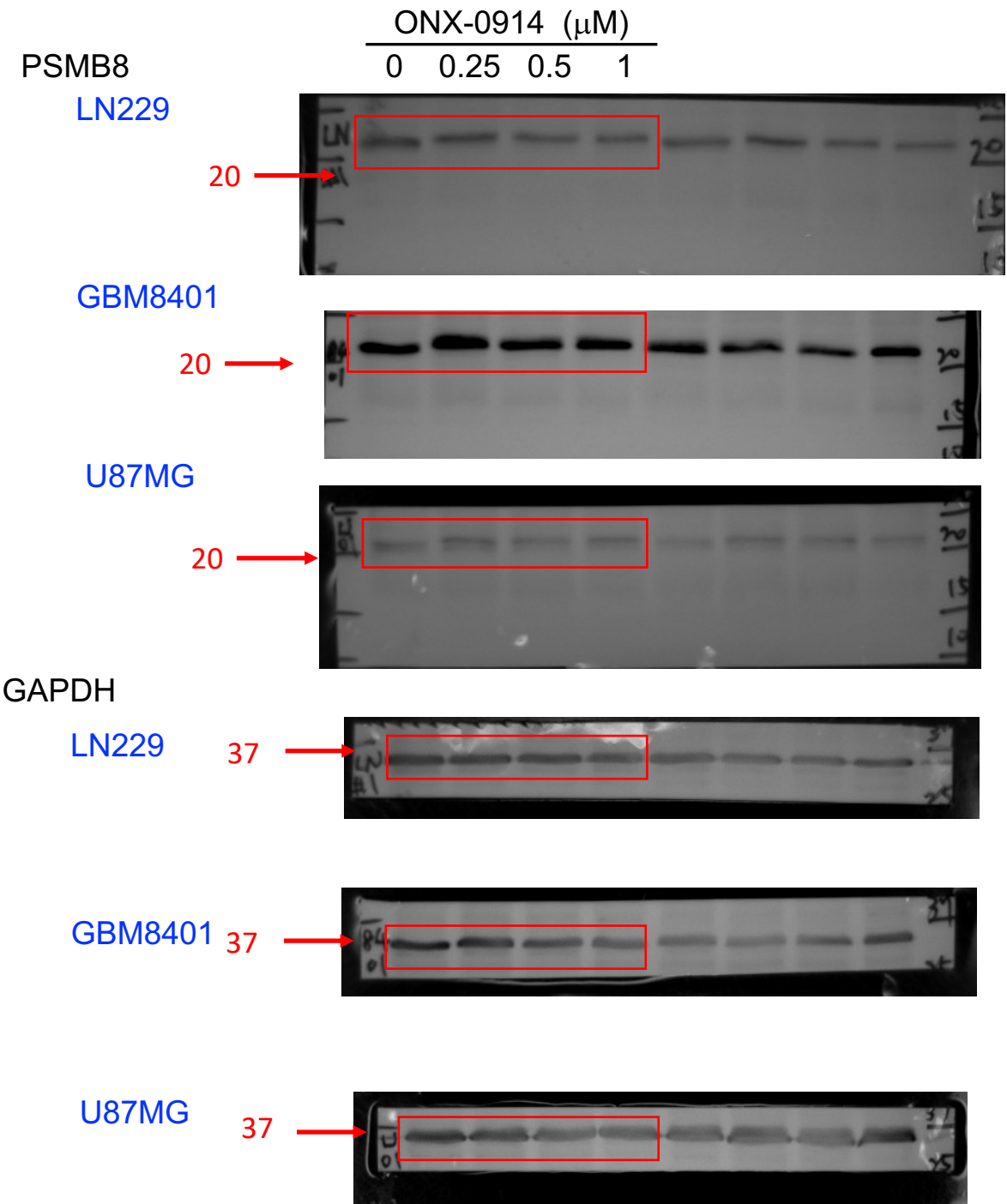

Marker MW  
Predict MW

Fig 4A

p-p53

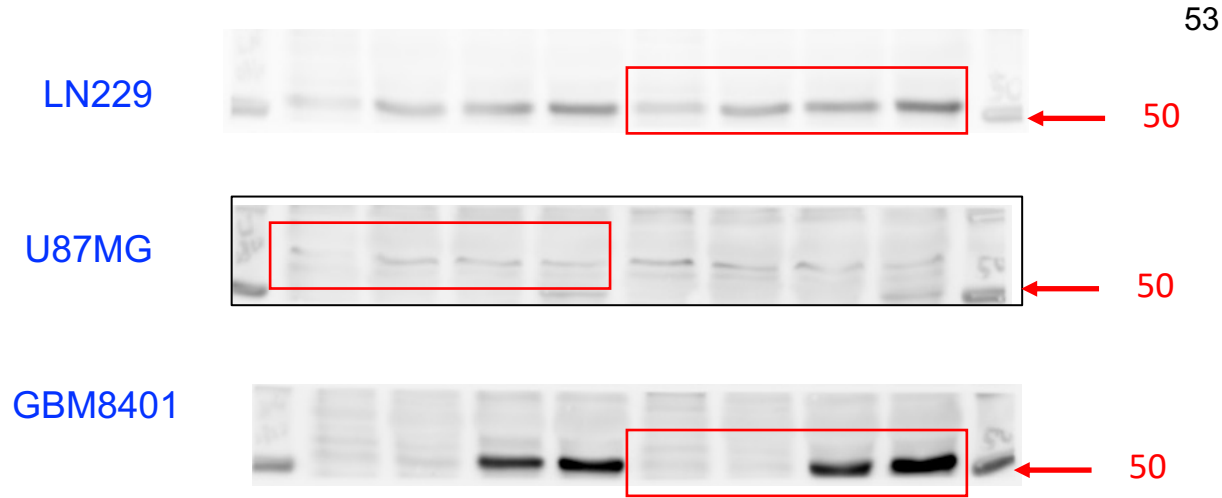

p53

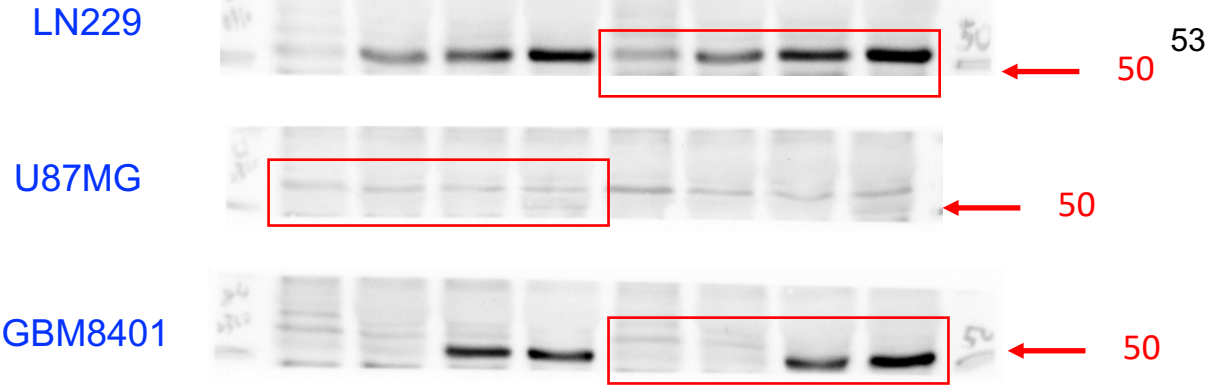

GAPDH

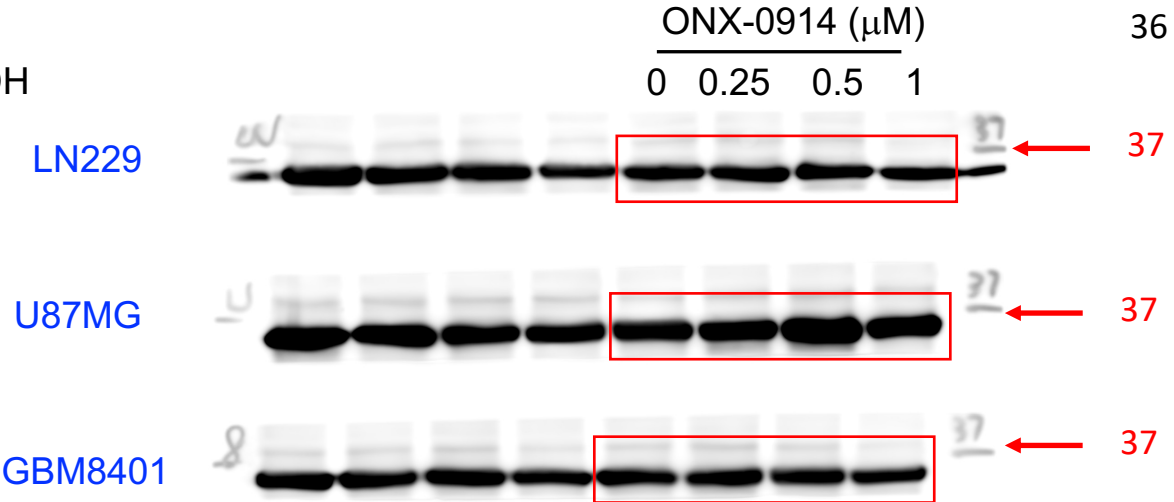

Fig 5D

Marker MW  
Predict MW

P62

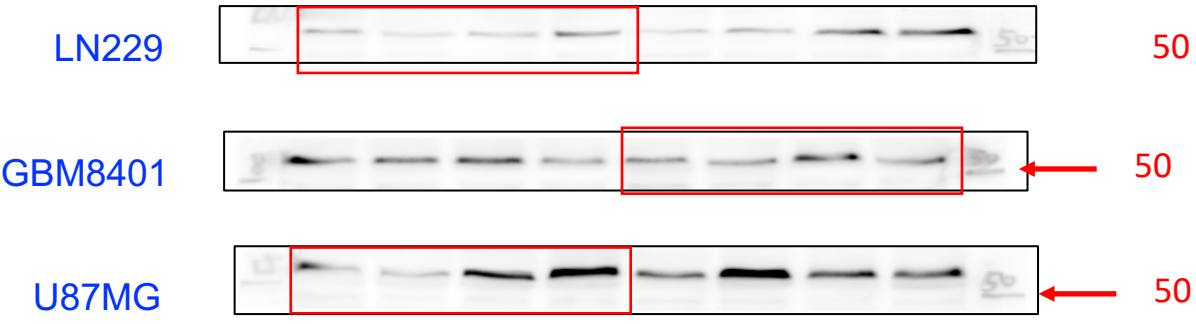

LC3B

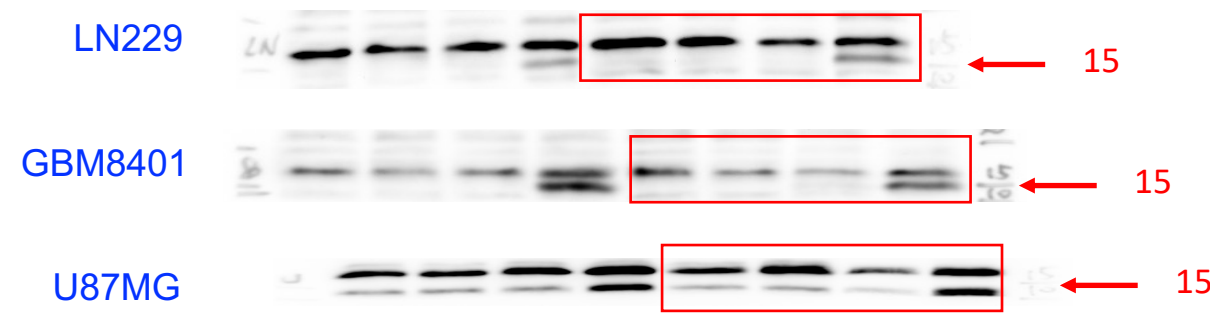

GAPDH

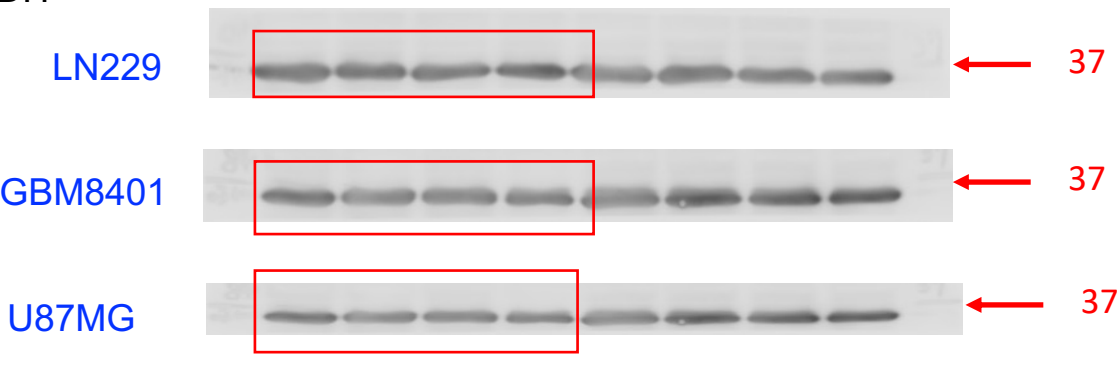

Supplement: Supplementary file 1 [file cancers-14-05712-s001.zip › File S1.pdf]
